# Primary and secondary care service use and costs associated with frailty in an ageing population: longitudinal analysis of an English primary care cohort of adults aged 50 and over, 2006–2017

**DOI:** 10.1093/ageing/afae010

**Published:** 2024-02-09

**Authors:** Carole Fogg, Tracey England, Shihua Zhu, Jeremy Jones, Simon de Lusignan, Simon D S Fraser, Paul Roderick, Andy Clegg, Scott Harris, Sally Brailsford, Abigail Barkham, Harnish P Patel, Bronagh Walsh

**Affiliations:** School of Health Sciences, Faculty of Environmental and Life Sciences, University of Southampton, Southampton, UK; School of Health Sciences, Faculty of Environmental and Life Sciences, University of Southampton, Southampton, UK; School of Primary Care, Population Sciences, and Medical Education, Faculty of Medicine, University of Southampton, Southampton, UK; School of Health Sciences, Faculty of Environmental and Life Sciences, University of Southampton, Southampton, UK; Nuffield Department of Primary Care Health Sciences, University of Oxford, Oxford, UK; School of Primary Care, Population Sciences, and Medical Education, Faculty of Medicine, University of Southampton, Southampton, UK; School of Primary Care, Population Sciences, and Medical Education, Faculty of Medicine, University of Southampton, Southampton, UK; Academic Unit for Ageing & Stroke Research, University of Leeds, Bradford Teaching Hospitals NHS Foundation Trust, Bradford, UK; School of Primary Care, Population Sciences, and Medical Education, Faculty of Medicine, University of Southampton, Southampton, UK; Southampton Business School, University of Southampton, Southampton, UK; Southern Health NHS Foundation Trust, Unit 1 Wessex Way, Colden Common, Winchester SO21 1WP, UK; University Hospitals Southampton NHS Foundation Trust, Southampton General Hospital, Southampton, UK; NIHR Southampton Biomedical Research Centre, Southampton Centre for Biomedical Research, Southampton, UK; School of Health Sciences, Faculty of Environmental and Life Sciences, University of Southampton, Southampton, UK

**Keywords:** frailty, health care costs, older people, primary health care, secondary health care

## Abstract

**Background:**

Frailty becomes more prevalent and healthcare needs increase with age. Information on the impact of frailty on population level use of health services and associated costs is needed to plan for ageing populations.

**Aim:**

To describe primary and secondary care service use and associated costs by electronic Frailty Index (eFI) category.

**Design and Setting:**

Retrospective cohort using electronic health records. Participants aged ≥50 registered in primary care practices contributing to the Oxford Royal College of General Practitioners Research and Surveillance Centre, 2006–2017.

**Methods:**

Primary and secondary care use (totals and means) were stratified by eFI category and age group. Standardised 2017 costs were used to calculate primary, secondary and overall costs. Generalised linear models explored associations between frailty, sociodemographic characteristics. Adjusted mean costs and cost ratios were produced.

**Results:**

Individual mean annual use of primary and secondary care services increased with increasing frailty severity. Overall cohort care costs for were highest in mild frailty in all 12 years, followed by moderate and severe, although the proportion of the population with severe frailty can be expected to increase over time. After adjusting for sociodemographic factors, compared to the fit category, individual annual costs doubled in mild frailty, tripled in moderate and quadrupled in severe.

**Conclusions:**

Increasing levels of frailty are associated with an additional burden of individual service use. However, individuals with mild and moderate frailty contribute to higher overall costs. Earlier intervention may have the most potential to reduce service use and costs at population level.

## Key Points

- Use of primary and secondary care services escalated with increasing frailty severity at all ages.
- After adjusting for age and other sociodemographic factors, frailty remained the main driver of service use and costs.
- Adjusted annual cost estimates doubled in people with mild frailty versus fit, trebled in moderate and quadrupled in severe frailty.
- Higher numbers of people with mild and moderate frailty led to higher population level costs as compared to severe frailty.
- Early, targeted intervention to prevent frailty onset and manage patient outcomes is key to reduce healthcare use and costs.

## Introduction

Frailty is a state of vulnerability associated with an increased risk of adverse health outcomes including hospital admission and mortality [1]. Frailty is associated with higher health and social care service use, and data on frailty prevalence is used by NHS England to guide commissioning of healthcare services. Since 2017, general practices in England are required to screen their populations for moderate/severe frailty and provide patients with appropriate interventions, e.g. medication reviews and falls risk assessments [2]. Analyses of health service use and costs have shown that increases in frailty are associated with incremental increases in costs beyond what would be predicted from multiple morbidity and disability [3, 4]. Associations between frailty and increased healthcare service use have previously been described, in general practice (GP) on-site appointments, remote consultations and physiotherapy contacts [5]; specialist outpatient clinic appointments, day surgery and emergency department visits [6]; help with meals or household duties, spending at least one night in a hospital or nursing home [7] and other community health and social care services [8, 9]. Longitudinal latent class analysis of a small ageing cohort in Ireland identified different classes of primary and secondary healthcare utilisation for community-dwelling older people which transitioned over time to reflect changes in healthcare need, the drivers of which need further exploration and for which frailty may be a key factor [10]. Additional healthcare utilisation with increasing frailty severity is in turn associated with higher costs [11, 12], mostly attributable to increases in hospital admissions and inpatient bed days.

Previous studies in this area have used phenotypic measures of frailty that would require additional resources to apply widely in practice or larger-scale research. Research is required to determine predicted costs for frail older people using larger, representative samples and frailty measures based on routine healthcare data. Additionally, previous research has focused on analyses at the individual level. Although the prevalence of moderate and severe frailty is greater in people aged ≥75, absolute numbers of people living with mild or moderate frailty in adults aged 50–74 are higher [13]. Understanding the impact of frailty across the age and frailty spectrum in the population in relation to use of community and acute healthcare services and related costs is key to inform planning appropriate services from middle age to the older population most at risk from adverse outcomes.

However, there is currently little available data describing how the distribution of frailty within the population impacts on a broad range of service use and costs at the population level over time. The aim of this analysis was to examine the impact of frailty on individual primary and secondary care service use and costs and to translate these findings to the larger population level to describe longitudinal trends in costs in a nationally representative cohort of people aged ≥50 in England.

## Methods

### Study design

Retrospective open cohort study using electronic health records from the Royal College of General Practitioners (RCGP) Research and Surveillance Centre (RSC) sentinel network, which collates routine primary care data from ˃500 GP practices in England and is nationally representative [14, 15].

### Population and sample size

Primary care patients aged ≥50 years, registered at a GP practice contributing to the RCGP RSC databank between 2006 and 2017 were eligible, including patients turning 50 or moving to a participating practice during this period. Patients left the cohort by moving out of participating practices or dying.

### Data description

The RCGP RSC databank provided data on patient sociodemographics, frailty, primary care service use and prescriptions. Pseudonymised patient records were linked to Hospital Episode Statistics secondary care data (NHS Digital) and mortality data (Office for National Statistics).

The 36-deficit electronic Frailty Index (eFI) score [16] was generated from the electronic patient primary care record on 1st January of each calendar year for each patient in the cohort, therefore the eFI data were complete. The eFI score was categorised: fit (0–0.12), mild (0.13–0.24), moderate (0.25–0.36) and severe (>0.36) [16]. Age was categorised into four groups (50–64, 65–74, 75–84, 85+). The 2015 Indices of Multiple Deprivation (IMD) quintiles were used [17]. The number of primary care contacts with a General Practitioner (face-to-face appointments, home visits, telephone appointments and e-consultations) for each participant for each year present in the cohort were calculated (maximum of 1 of each contact type per day). The total number of medications (sum of individual medications*number of times prescribed) per participant per year was calculated. Annual individual visits to emergency departments, outpatient appointments, hospital admissions, length of hospital stay and critical care (CC) admissions were calculated.

### Statistical and cost analysis

Total service use and summary statistics for each calendar year (2006–2017) were calculated. Annual means and standard deviations for each service use component in primary and secondary care were summarised by combinations of frailty category and age group.

Costs were calculated per patient/year using unit costs from 2016/2017 NHS National Reference costs (18) and Personal Social Services Research Unit (PSSRU) Unit Costs of Health and Social Care data 2017, or the nearest calendar year if unavailable in 2017, adjusting prices to the 2017 base [19–21]. Primary care unit costs used were: GP face-to-face visit £38 [20]; GP home visit £75.84, estimated at 12.8 min including travel time [19]; telephone appointment £36 [20]; e-consultation £37.27 [21]; cost per medication prescribed £8.20 [22]. Secondary care costs used were: outpatient appointment £138 [20]; Emergency department visit £151.50; hospital admissions of ≥24 h £384 per day, hospital stay <24 h £322; critical care admission £1,082.30 [18]. Total costs for each service use component and a summary cost for primary, secondary and total care (primary + secondary) were calculated per patient/year. Average annual costs were calculated for each frailty category by age group (sum of costs per age group/frailty category for contributing calendar years divided by total number of calendar years). Generalised linear models using a gamma distribution explored the association between frailty and primary, secondary and total care costs, adjusted by key variables identified in previous work (age group, sex, ethnicity, deprivation and urban/rural location [23]) presented with 95% confidence intervals (CI). A Gamma error distribution with a log link provided the best fit due to the shape of the cost data. Adjusted annual mean costs for each frailty category and cost ratios (‘fit’ category as baseline) were generated. Summative costs per year/frailty category for the whole cohort were plotted.

### Ethics

The study was approved by the University of Southampton Research Ethics Committee (ref 46,313) on 6/2/2019, the RCGP RSC Joint Research and Surveillance Centre Committee on 24/1/2019 and the NHS Digital DARS IGARD panel on 19/4/2021.

## Results

### Cohort description

The cohort comprised 2,171,497 patients from 419 GP practices—1,104,135 patients in 2006 rising to 1,489,495 in 2017—and 15,514,734 person-years of data [23]. Frailty prevalence increased from 26.5% to 38.9% from 2006 to 2017. Mild frailty was most common, increasing from 20.5% in 2006 to 25.3% in 2017, ranging from 16.5% of people aged 50–64 to 39.2% of people aged 75–84 in 2017. People aged ≥85 had the highest prevalence of moderate and severe frailty (33.3% and 24.4%, respectively, in 2017). Additional cohort details, including the number and age composition of the cohort by calendar year, are presented elsewhere [13, 23].

### Primary care service use

Mean annual use of each primary care service type increased with levels of frailty (Table 1), a pattern that was also observed when stratified by age group. Face-to-face appointments were slightly higher in the oldest groups across frailty categories, whereas home visits were slightly higher for this group in all frailty categories. Overall use of most services increased over the study period as the population increased and aged, for example face-to-face visits increased from >7.3 million in 2006 to >9.5 million in 2017, prescriptions from >28 million to >52 million and telephone triage from >500,000 to >1.9 million (Appendix 1). Home visits were the only service to decrease, from >380,000 to >319,000.

**Table 1:** Summary statistics of primary care service use by frailty category and age group

Frailty category *Age group*	Number of contributing calendar years^a^	Type of primary care service use				
		Face-to-face appointments Mean (SD)	Home visits Mean (SD)	Telephone triage Mean (SD)	e-consultations Mean (SD)	Number of individual prescriptions for medicines Mean (SD)
**Overall**						
**Fit**	10,143,679	4.9 (6.9)	0.066 (0.73)	0.52 (1.4)	0.0025 (0.095)	13.9 (21.9)
**Mild**	3,707,666	9.9 (10.9)	0.38 (2.01)	1.3 (2.6)	0.0045 (0.14)	52.0 (45.8)
**Moderate**	1,254,796	12.2 (13.7)	1.1 (3.7)	2.3 (3.9)	0.0056 (0.17)	86.9 (73.2)
**Severe**	408,593	13.2 (15.8)	2.3 (5.6)	3.7 (5.6)	0.0078 (0.21)	131.1 (109.5)
**Fit**						
***50–64***	6,697,966	4.3 (6.2)	0.029 (0.47)	0.47 (1.3)	0.0025 (0.095)	10.9 (19.9)
***65–74***	2,397,527	5.9 (7.5)	0.064 (0.71)	0.56 (1.5)	0.0028 (0.10)	18.6 (22.9)
***75–84***	858,897	6.6 (8.4)	0.20 (1.3)	0.70 (1.8)	0.0019 (0.086)	22.2 (25.6)
***85+***	189,289	5.9 (9.0)	0.76 (2.5)	0.92 (2.17)	0.0013 (0.050)	23.5 (30.6)
**Mild**						
***50–64***	1,108,641	9.5 (10.5)	0.13 (1.2)	1.3 (2.6)	0.0058 (0.15)	48.6 (49.5)
***65–74***	1,210,346	10.1 (10.8)	0.20 (1.5)	1.2 (2.4)	0.0051 (0.15)	52.3 (41.9)
***75–84***	1,011,372	10.4 (11.4)	0.50 (2.3)	1.4 (2.7)	0.0032 (0.12)	53.9 (43.5)
***85+***	377,307	8.9 (11.5)	1.4 (3.8)	1.7 (3.1)	0.0024 (0.11)	56.1 (51.2)
**Moderate**						
***50–64***	152,409	13.2 (13.8)	0.37 (2.3)	2.3 (4.4)	0.0091 (0.21)	95.4 (90.1)
***65–74***	296,118	13.3 (13.9)	0.52 (3.0)	2.1 (3.8)	0.0075 (0.19)	88.5 (71.6)
***75–84***	487,797	12.7 (13.8)	0.98 (3.5)	2.2 (3.8)	0.0047 (0.16)	85.2 (69.1)
***85+***	318,472	10.0 (13.0)	2.0 (4.7)	2.5 (4.0)	0.0037 (0.13)	84.1 (71.3)
**Severe**						
***50–64***	20,138	17.1 (18.1)	0.97 (4.0)	4.3 (7.3)	0.022 (0.38)	162.6 (143.7)
***65–74***	55,727	16.3 (17.2)	1.2 (4.4)	3.8 (5.8)	0.015 (0.32)	142.8 (114.8)
***75–84***	159,707	14.4 (16.1)	2.0 (5.6)	3.6 (5.5)	0.0065 (0.19)	131.7 (109.0)
***85+***	173,021	10.6 (14.3)	3.0 (6.0)	3.7 (5.3)	0.0050 (0.14)	123.2 (102.3)

^a^The number of person-years of follow-up contributed to the cohort by people in the respective age/frailty categories as of 1st January for each calendar year.

### Secondary care service use

Mean annual use of all secondary care services increased with frailty severity (Table 2), a trend that was also observed within age groups. Mean annual outpatient appointments, Emergency Department (ED) attendances and hospital admissions for patients with mild frailty were similar across age groups. The younger age groups had higher average outpatient appointments and elective hospital admissions for those with moderate and severe frailty, in contrast to unplanned admissions, which increased with age, other than in severe frailty which was comparable across ages.

**Table 2:** Summary statistics of secondary care service use by frailty category and age group

Frailty category *Age group*	Number of contributing years^a^		Type of secondary care service use							
				Hospital admissions				Days of hospital stay		
		Outpatient appointments Mean (SD)	Emergency department attendances Mean (SD)	Total Mean (SD)	Elective Mean (SD)	Unplanned Mean (SD)	Critical care	Total^b^ Mean (SD)	Elective^c^ Mean (SD)	Unplanned^c^ Mean (SD)
**Fit**	10,143,679	1.4 (3.5)	0.15 (0.59)	0.27 (1.4)	0.20 (1.3)	0.07 (0.35)	0.0027 (0.054)	4.6 (18.9)	1.1 (9.3)	3.1 (14.0)
**Mild**	3,707,666	3.4 (5.5)	0.32 (0.89)	0.66 (3.1)	0.45 (3.0)	0.21 (0.66)	0.0071 (0.088)	7.5 (22.0)	1.4 (10.5)	5.5 (16.4)
**Moderate**	1,254,796	4.8 (6.9)	0.57 (1.2)	1.1 (5.1)	0.64 (4.9)	0.42 (0.95)	0.011 (0.11)	11.5 (24.4)	1.4 (9.6)	9.1 (19.8)
**Severe**	408,593	5.8 (9.2)	0.92 (1.6)	1.5 (6.0)	0.71 (5.8)	0.75 (1.3)	0.013 (0.12)	16.3 (27.8)	1.3 (9.6)	13.8 (23.5)
**Fit**										
***50–64***	6,697,966	1.3 (3.3)	0.15 (0.59)	0.22 (1.3)	0.17 (1.2)	0.05 (0.31)	0.0020 (0.047)	3.3 (17.3)	0.96 (8.2)	2.1 (12.7)
***65–74***	2,397,527	1.7 (3.8)	0.14 (0.54)	0.33 (1.6)	0.25 (1.5)	0.07 (0.36)	0.0037 (0.064)	4.6 (18.3)	1.3 (10.8)	2.9 (12.8)
***75–84***	858,897	2.0 (3.9)	0.18 (0.60)	0.40 (1.7)	0.27 (1.5)	0.12 (0.46)	0.0045 (0.069)	7.7 (22.4)	1.4 (10.4)	5.5 (17.6)
***85+***	189,289	1.7 (3.5)	0.28 (0.77)	0.44 (1.6)	0.18 (1.4)	0.25 (0.64)	0.0027 (0.052)	14.4 (28.0)	1.2 (9.8)	11.6 (23.1)
**Mild**										
***50–64***	1,108,641	3.5 (5.8)	0.33 (0.97)	0.62 (3.5)	0.46 (3.4)	0.16 (0.65)	0.0066 (0.086)	4.9 (20.1)	1.2 (10.8)	3.3 (14.4)
***65–74***	1,210,346	3.5 (5.5)	0.28 (0.82)	0.67 (3.1)	0.49 (3.0)	0.17 (0.60)	0.0079 (0.093)	5.8 (21.8)	1.4 (12.1)	4.0 (15.2)
***75–84***	1,011,372	3.4 (5.2)	0.32 (0.83)	0.70 (3.0)	0.46 (2.9)	0.24 (0.66)	0.0077 (0.091)	8.7 (21.7)	1.4 (8.8)	6.5 (16.9)
***85+***	377,307	2.7 (4.6)	0.45 (0.99)	0.67 (2.2)	0.27 (2.0)	0.38 (0.80)	0.0041 (0.065)	14.6 (25.2)	1.2 (8.6)	11.8 (20.6)
**Moderate**										
***50–64***	152,409	6.2 (8.6)	0.62 (1.6)	1.3 (7.3)	0.94 (7.1)	0.36 (1.0)	0.015 (0.13)	7.4 (21.8)	1.4 (8.1)	5.6 (17.6)
***65–74***	296,118	5.5 (7.6)	0.50 (1.2)	1.2 (5.8)	0.82 (5.6)	0.35 (0.92)	0.015 (0.13)	8.5 (22.1)	1.5 (9.0)	6.3 (17.8)
***75–84***	487,797	4.8 (6.5)	0.54 (1.1)	1.1 (4.8)	0.65 (4.7)	0.42 (0.92)	0.012 (0.11)	11.4 (24.8)	1.5 (10.9)	8.9 (19.7)
***85+***	318,472	3.4 (5.8)	0.64 (1.2)	0.89 (2.9)	0.32 (2.7)	0.54 (0.98)	0.0053 (0.074)	16.2 (26.1)	1.09 (8.6)	13.5 (21.7)
**Severe**										
***50–64***	20,138	9.8 (11.6)	1.1 (2.4)	2.6 (11.9)	1.8 (11.7)	0.75 (1.7)	0.028 (0.19)	11.6 (26.8)	1.7 (9.9)	9.1 (21.9)
***65–74***	55,727	8.2 (10.4)	0.9 (1.7)	1.9 (8.1)	1.2 (7.9)	0.71 (1.4)	0.023 (0.16)	12.9 (26.7)	1.5 (8.5)	10.5 (22.6)
***75–84***	159,707	6.3 (9.4)	0.90 (1.6)	1.5 (6.1)	0.79 (5.9)	0.73 (1.3)	0.015 (0.12)	15.6 (28.1)	1.4 (10.1)	13.0 (23.8)
***85+***	173,021	4.1 (7.8)	0.92 (1.5)	1.2 (3.7)	0.36 (3.4)	0.77 (1.2)	0.0062 (0.080)	18.8 (27.7)	1.0 (9.4)	16.2 (23.3)

^a^The number of person-years of follow-up contributed to the cohort by people in the respective age/frailty categories as of 1st January for each calendar year.

^b^In the calendar years where the patient had a hospital admission.

^c^In the calendar years where the patient had an elective admission or an unplanned admission.

Unplanned care and total bed days increased with frailty severity. This trend was observed within age groups, other than for the oldest group, where these were more similar across frailty categories. However, total days hospitalised increased with frailty severity, but within age groups, the largest difference was observed in the 50–64 group (3 days to 11 days) compared with 14 days for fit patients to 18 days for those with severe frailty in the 85+ group. People with severe frailty of any age had comparable mean hospitalisation days (between 11 and 18 days). Total cohort outpatient appointments increased during the study period from >1.8 million (2006) to >4.2 million (2017), with a large increase also in A&E from >151,000 to >470,000 (Appendix 2). Total admissions increased from >429,000 to >740,000, but total days of hospital stay varied less, ranging between 1.9 and 2.1 million each year.

### Cost of services

Annual overall costs for the whole cohort (sum of total care costs for each category in a calendar year) increased from £1.65 billion in 2006 to £2.5 billion in 2017 (Table 3). Total costs for all frailty categories increased over the cohort period as the size of the cohort increased and the population aged and became more frail. Although individual mean costs for people living with severe frailty are highest, the large numbers of people living with mild and moderate frailty results in larger overall total care costs in these frailty categories. When considered as a proportion of the total costs for the cohort, between 33% and 36% were attributed to people with mild frailty in different calendar years and between 15% and 23% with moderate frailty (Table 3, Appendix 3). The severe frailty group represented only 5% of the overall total care costs in 2006 but increased throughout the study period to 15% in 2017, with similar patterns of increase in primary and secondary care costs. Conversely, as cohort participants aged and transitioned to more severe categories of frailty, the proportion of annual total care costs incurred by people in the fit category decreased from 44% to 29% in 2017.

**Table 3:** Primary, secondary and total care costs for the whole cohort by frailty category

Calendar year	Number of patients	Primary care total costs £	%	Secondary care total costs £	%	Total care costs £	%
**2006**							
*Fit*	811,384	270,913,810	48%	460,068,822	43%	730,982,632	44%
*Mild*	225,818	203,622,163	36%	391,478,306	36%	595,100,469	36%
*Moderate*	55,319	72,554,916	13%	173,939,200	16%	246,494,116	15%
*Severe*	11,614	20,265,358	4%	55,713,752	5%	75,979,110	5%
***Total***	*1,104,135*	*567,356,250*		*1,081,200,080*		*1,648,556,329*	
**2007**							
*Fit*	808,706	267,202,515	44%	436,043,591	39%	703,246,106	41%
*Mild*	245,213	220,042,701	36%	406,754,525	36%	626,797,226	36%
*Moderate*	67,501	88,405,201	15%	199,874,111	18%	288,279,311	17%
*Severe*	16,163	28,768,221	5%	72,683,752	7%	101,451,973	6%
***Total***	*1,137,583*	*604,418,638*		*1,115,355,979*		*1,719,774,616*	
**2008**							
*Fit*	809,935	269,874,025	42%	442,764,661	37%	712,638,685	39%
*Mild*	261,829	236,677,808	37%	432,873,140	36%	669,550,948	36%
*Moderate*	77,273	102,646,675	16%	229,690,823	19%	332,337,498	18%
*Severe*	19,918	36,016,479	6%	91,447,929	8%	127,464,408	7%
***Total***	*1,168,955*	*645,214,987*		*1,196,776,553*		*1,841,991,539*	
**2009**							
*Fit*	812,544	278,752,429	40%	449,165,119	35%	727,917,548	37%
*Mild*	276,994	255,481,622	37%	451,692,877	36%	707,174,500	36%
*Moderate*	85,936	117,001,731	17%	261,252,083	21%	378,253,815	19%
*Severe*	23,315	42,993,942	6%	109,937,157	9%	152,931,100	8%
***Total***	*1,198,789*	*694,229,726*		*1,272,047,238*		*1,966,276,964*	
**2010**							
*Fit*	817,062	275,176,182	38%	432,000,398	33%	707,176,580	35%
*Mild*	291,974	265,669,782	37%	456,099,858	35%	721,769,641	36%
*Moderate*	95,308	130,391,431	18%	281,784,103	22%	412,175,534	20%
*Severe*	27,262	50,736,367	7%	126,058,761	10%	176,795,129	9%
***Total***	*1,231,606*	*721,973,762*		*1,295,943,120*		*2,017,916,884*	
**2011**							
*Fit*	826,307	275,912,500	37%	428,257,931	33%	704,170,431	34%
*Mild*	306,064	275,431,399	37%	456,203,478	35%	731,634,877	35%
*Moderate*	103,329	141,151,491	19%	289,219,413	22%	430,370,904	21%
*Severe*	31,047	58,908,732	8%	138,334,424	11%	197,243,156	10%
***Total***	*1,266,747*	*751,404,122*		*1,312,015,246*		*2,063,419,368*	
**2012**							
*Fit*	837,860	276,768,142	36%	435,431,292	31%	712,199,435	33%
*Mild*	318,786	283,740,737	36%	478,616,430	34%	762,357,167	35%
*Moderate*	110,930	151,014,414	19%	315,436,358	23%	466,450,772	22%
*Severe*	35,239	66,711,080	9%	160,782,620	12%	227,493,700	10%
***Total***	*1,302,815*	*778,234,374*		*1,390,266,702*		*2,168,501,076*	
**2013**							
*Fit*	854,958	274,641,735	34%	447,799,261	31%	722,440,995	32%
*Mild*	333,258	290,805,633	36%	493,391,511	34%	789,197,144	35%
*Moderate*	118,436	159,564,086	20%	332,863,148	23%	492,427,235	22%
*Severe*	39,275	74,530,775	9%	174,738,641	12%	249,269,416	11%
***Total***	*1,345,927*	*799,542,229*		*1,448,792,561*		*2,253,334,790*	
**2014**							
*Fit*	867,579	272,245,970	33%	463,275,367	30%	735,521,337	31%
*Mild*	345,492	298,169,597	36%	515,626,969	34%	813,796,565	34%
*Moderate*	125,367	169,159,965	21%	356,379,561	23%	525,539,527	22%
*Severe*	43,582	83,752,657	10%	200,249,359	13%	284,002,016	12%
***Total***	*1,382,020*	*823,328,189*		*1,535,531,256*		*2,358,859,445*	
**2015**							
*Fit*	886,165	273,093,745	32%	471,317,545	30%	744,411,291	31%
*Mild*	358,064	303,570,983	36%	525,418,773	33%	828,989,756	34%
*Moderate*	132,375	176,226,724	21%	371,645,799	23%	547,872,523	22%
*Severe*	48,671	92,960,048	11%	222,490,129	14%	315,450,177	13%
***Total***	*1,425,275*	*845,851,500*		*1,590,872,248*		*2,436,723,748*	
**2016**							
*Fit*	901,512	275,672,502	32%	475,153,546	28%	750,826,048	30%
*Mild*	367,611	308,255,836	35%	545,368,330	33%	853,624,166	34%
*Moderate*	138,603	182,678,697	21%	388,561,190	23%	571,219,886	23%
*Severe*	53,661	101,894,602	12%	268,839,942	16%	356,986,812	14%
***Total***	*1,461,387*	*868,501,637*		*1,677,923,008*		*2,532,656,912*	
**2017**							
*Fit*	909,667	275,606,753	31%	464,879,024	28%	740,485,777	29%
*Mild*	376,563	312,259,546	35%	526,305,782	32%	838,565,328	33%
*Moderate*	144,419	189,752,440	21%	389,353,489	24%	579,105,930	23%
*Severe*	58,846	111,631,201	13%	268,839,942	16%	380,471,144	15%
***Total***	*1,489,495*	*889,249,942*		*1,649,378,238*		*2,538,628,180*	

Mean primary care annual costs tripled in mild frailty compared to fit and increased 4-fold and 5-fold in moderate and severe frailty, respectively (Table 4). Stratification of frailty groups by age revealed unexpected patterns of service use and costs. Those aged 50–64 showed the expected increase in costs with severity of frailty, partly due to the lower cost for ‘fit’ people, but also due to this age group having the highest costs when severely frail, whereas the oldest ages had the lowest costs in moderate and severe frailty. In contrast, the 85+ age group had the highest annual secondary care costs in mild and moderate categories and costs decreased with decreasing age, except for the severely frail patients which had similar costs across all ages. Increased cost with severity was mostly driven by higher hospitalisation costs in patients aged 50–74 with moderate/severe frailty.

**Table 4:** Average annual primary, secondary (also elective and unplanned costs) and total care costs by age and frailty category (descriptive costs)

	Primary care costs £ Mean (SD)	Secondary care costs £ Mean (SD)	Elective costs £ Mean (SD)	Unplanned costs £ Mean (SD)	Total care costs £ Mean (SD)
**Overall**					
**Fit**	324 (388)	533 (3,017)	115 (1,444)	173 (2,072)	857 (3,105)
**Mild**	878 (665)	1,533 (5,211)	280 (2,457)	652 (3,626)	2,411 (5,355)
**Moderate**	1,339 (936)	2,861 (7,076)	409 (2,952)	1,531 (5,295)	4,200 (7,275)
**Severe**	1,882 (1,272)	4,592 (9,072)	468 (3,356)	2,900 (7,155)	6,475 (9,344)
**Frailty category** ***Age group***					
**Fit**					
***50–64***	271 (349)	407 (2570)	90 (1,199)	105 (1,724)	678 (2,649)
***65–74***	402 (407)	624 (3190)	152 (1,782)	190 (2,045)	1,027 (3,280)
***75–84***	476 (466)	997 (4330)	188 (1,921)	447 (3,027)	1,473 (4,423)
***85+***	510 (561)	1,740 (6080)	164 (1,955)	1,137 (4,867)	2,250 (6,174)
**Mild**					
***50–64***	813 (666)	1,190 (4,598)	256 (2,469)	360 (2,955)	2,003 (4,752)
***65–74***	869 (624)	1,343 (5,065)	295 (2,752)	469 (3,262)	2,212 (5,198)
***75–84***	925 (669)	1,765 (5,372)	306 (2,181)	828 (3,901)	2,689 (5,515)
***85+***	966 (752)	2,531 (6,595)	236 (2,087)	1,632 (5,214)	3,498 (6,724)
**Moderate**					
***50–64***	1,396 (1,046)	2,446 (6,628)	485 (3,127)	917 (4,496)	3,842 (6,889)
***65–74***	1,347 (929)	2,494 (6,523)	480 (2,976)	1,061 (4,636)	3,841 (6,750)
***75–84***	1,334 (910)	2,868 (7,163)	431 (3,202)	1,520 (5,295)	4,202 (7,356)
***85+***	1,313 (923)	3,390 (7,591)	271 (2,386)	2,278 (6,078)	4,704 (7,757)
**Severe**					
***50–64***	2,214 (1,568)	4,656 (9,909)	908 (4,950)	2,055 (6,612)	6,870 (10,267)
***65–74***	2,021 (1,323)	4,494 (9,103)	679 (3,631)	2,333 (6,858)	6,515 (9,421)
***75–84***	1,910 (1,282)	4,592 (9,176)	520 (3,506)	2,800 (7,265)	6,503 (9,466)
***85+***	1,774 (1,193)	4,617 (8,862)	301 (2,844)	3,274 (7,184)	6,390 (9,089)
**All frailty categories**					
***50–64***	372 (506)	565 (3,124)	123 (1520)	161 (2,057)	938 (3,266)
***65–74***	638 (650)	1,038 (4,337)	228 (2,258)	371 (2,858)	1,677 (4,522)
***75–84***	913 (825)	1,896 (5,848)	303 (2,438)	957 (4,333)	2,809 (6,076)
***85+***	1,121 (950)	2,989 (7,290)	244 (2,297)	2,006 (5,828)	4,111 (7,501)

Adjusted analyses showed that frailty was the main driver of primary, secondary and total care costs (Table 5). After frailty, the most important influences on costs were age and deprivation, with minor associations with ethnicity, sex and location. The adjusted predicted mean costs for each frailty category, doubled in patients with mild frailty, tripled in moderate and more than quadrupled in severe (Appendix 4). For total care costs, compared to a person in the ‘fit’ category, this translates to an additional £1,201 for people with mild frailty, £2,262 for moderate frailty and £3,507 for severe frailty.

**Table 5:** Association of frailty and sociodemographic factors with costs

	Primary care			Secondary care			Total care		
	Coefficient	*P*-value	Ratio of mean costs [95% CI]	Coefficient	*P*-value	Ratio of mean costs [95% CI]	Coefficient	*P*-value	Ratio of mean costs [95% CI]
**Frailty category**									
Fit	–	–	–	–	–	–	–	–	–
Mild	0.850	<0.001	2.34 [2.34–2.34]	0.79	<0.001	2.21 [2.20–2.23]	0.813	<0.001	2.26 [2.25–2.26]
Moderate	1.19	<0.001	3.28 [3.27–3.29]	1.23	<0.001	3.42 [3.38–3.45]	1.21	<0.001	3.36 [3.34–3.38]
Severe	1.48	<0.001	4.38 [4.36–4.40]	1.57	<0.001	4.81 [4.73–4.89]	1.54	<0.001	4.66 [4.62–4.71]
**Age group**									
50–64	–	–	–	–	–	–	–	–	–
65–74	0.312	<0.001	1.37 [1.36–1.37]	0.378	<0.001	1.46 [1.45–1.47]	0.349	<0.001	1.42 [1.41–1.42]
75–84	0.387	<0.001	1.47 [1.47–1.48]	0.734	<0.001	2.08 [2.07–2.10]	0.600	<0.001	1.82 [1.81–1.82]
85+	0.395	<0.001	1.48 [1.48–1.49]	1.06	<0.001	2.88 [2.85–2.92]	0.821	<0.001	2.27 [2.26–2.29]
**Sex**									
Male	–	–	–	–	–	–	–	–	–
Female	0.084	<0.001	1.09 [1.09–1.09]	−0.065	<0.001	0.94 [0.93–0.94]	−0.009	<0.001	0.991 [0.988–0.995]
**IMD quintile**									
Least deprived	–	–	–	–	–	–	–	–	–
4th quintile	0.074	<0.001	1.08 [1.07–1.08]	0.060	<0.001	1.06 [1.05–1.07]	0.064	<0.001	1.07 [1.06–1.07]
3rd quintile	0.099	<0.001	1.10 [1.10–1.11]	0.124	<0.001	1.13 [1.12–1.14]	0.113	<0.001	1.12 [1.11–1.12]
2nd quintile	0.145	<0.001	1.16 [1.15–1.16]	0.219	<0.001	1.24 [1.23–1.25]	0.190	<0.001	1.21 [1.20–1.22]
Most deprived	0.211	<0.001	1.24 [1.23–1.24]	0.327	<0.001	1.39 [1.37–1.40]	0.284	<0.001	1.33 [1.32–1.34]
**Ethnicity**									
White	–	–	–	–	–	–	–	–	–
Asian	0.033	<0.001	1.03 [1.03–1.04]	−0.115	<0.001	0.891 [0.878–0.905]	−0.061	<0.001	0.94 [0.93–0.95]
Black	−0.086	<0.001	0.92 [0.91–0.92]	0.127	<0.001	1.14 [1.11–1.16]	0.051	<0.001	1.05 [1.04–1.07]
Mixed/Other	−0.115	<0.001	0.89 [0.89–0.90]	0.086	<0.001	1.09 [1.06–1.12]	0.013	0.131	1.01 [1.00–1.03]
Missing	−0.872	<0.001	0.42 [0.42–0.42]	−1.85	<0.001	0.16 [0.16–0.16]	−1.36	<0.001	0.26 [0.25–0.26]
**Rural/urban**									
Rural	–	–	–	–	–	–	–	–	–
Urban	−0.112	<0.001	0.89 [0.89–0.90]	0.045	<0.001	1.05 [1.04–1.05]	−0.017	<0.001	0.98 [0.98–0.99]

## Discussion

Our study confirms that people with moderate and severe frailty of all ages, measured by a frailty index tool using routine healthcare data, have greater use of healthcare services and higher associated costs than those who are fit or have mild frailty, and that this effect is independent of age and other socio-demographic factors. As frailty prevalence increased within the ageing cohort over the 12-year follow-up period [23], associated overall service use and costs also increased. Service providers and planners can expect to see growing numbers of older people living with frailty as populations age, with implications for projected cost impacts over the mid-term. Importantly, our analyses also show that despite lower individual costs, overall costs at population level are highest in mild and moderate frailty, due to the larger numbers of people in these groups. However, given global ageing, future shifts towards large absolute numbers with severe frailty are also anticipated, with further cost increases, particularly related to unplanned admissions. In keeping with findings elsewhere, sociodemographic factors including age, deprivation, ethnicity and gender, were independent predictors of service use and costs [12, 24], but frailty severity had the strongest association with service use and costs. The increased individual-level primary and secondary healthcare service use and costs associated with frailty should therefore be considered within the context of the wider demographic structure, particularly absolute numbers with mild and moderate frailty.

Care costs increased with frailty severity in all age groups, suggesting identification of people for frailty prevention and proactive management to reduce frailty progression are key to reduce future costs to the health service as the overall population ages, even within people in middle age. The embedding of routine-data based frailty index measures in practice in the UK and elsewhere will facilitate such interventions. In addition, this study adds important information about the population level impact of frailty. The large overall number of people in the population with mild and moderate frailty, and the length of time they live with frailty, is an important driver of overall costs and necessitates development of more cost-efficient services to manage support and care. Development of frailty at earlier ages in some groups, particularly those with higher levels of deprivation who are likely to have earlier onset [23], suggests targeted services could be beneficial as younger groups with severe frailty appear to have higher costs compared with older people. In contrast to previous observations regarding the inverse care law [25], our data suggest people living in more deprived areas are accessing more care; however, this trend does not mean that all care needs are being met [26]. The associations of other sociodemographic factors with frailty, service use and hence overall costs, needs better use of routinely available information to map geographical variability in need, to enable matching of funding for services more closely with the morbidity burden [27].

These analyses suggest the concept of frailty as a vulnerability, as measured by the eFI and similar tools, is reflected in service use as expected and that patterns of service use and costs are in line with those reported for phenotypic frailty measures. However, the pattern of associated costs at population level necessitates reframing of the response to frailty to focus not just on the oldest adults who have higher levels of service use regardless of their frailty status, but also on the large number of mid-aged and ‘younger-old’ adults who are already experiencing increased need for health services, which continue to accumulate as frailty progresses. Although multimorbidity has been a larger focus in mid-aged adults than frailty, the much higher costs in mid-aged adults with severe frailty as compared to the oldest adults requires a greater understanding of profiles of multimorbidity and how these interact with frailty and sociodemographic risk factors, including education and wealth, and the subsequent patient trajectories [28–31].

The estimates of adjusted costs for different levels of frailty facilitate prediction of future costs when combined with information on demographic trends, evidence on frailty trajectories and projections of disease burden [23, 32]. These predictions can be generated at local or regional using techniques such as simulation modelling to provide large-scale projections of trends in frailty and its associated service use and costs, and impact of different interventions can be explored [33]. For example, modifications in service design to reduce service use in people with frailty may be considered, including addressing gaps in care co-ordination for people living with frailty [34] and improving effective primary care (e.g. timely access, access to named practitioner, co-ordination with specialist services) [35] to reduce preventable adverse events, e.g. ED attendances and unplanned hospital admissions. Best practice management for people living with frailty includes a wide range of health and care services, with a particular focus on the importance of integrated care and personalised care support planning [36, 37]. However, our results suggest that attention should also be focussed on preventing or delaying frailty onset, particularly in mid-aged adults, to reduce future costs. Reducing service use requirements by reducing frailty prevalence and progression requires a strong public health approach with advice and interventions targeted across the life span and particularly in mid-life, in addition to proactive management of those living with frailty [38, 39]. This has implications not just for service design, but also the workforce required to deliver these changes, e.g. delivery by multi-professional teams including social prescribers and is the key to shaping the health of our future older population.

### Limitations

Older people are more likely to receive community health and social care services that were not available in our data sources, but which might explain reductions in GP face-to-face appointments and hospital outpatient visits with older age. These analyses might therefore under-estimate total care needs in the oldest age groups. More complete data are needed to have a whole-systems view of care provided to inform future commissioning of appropriate services for frail older people. Whereas the average service use and costs in the UK reflect a fairly standardised system of care, application to other settings will depend on the health system involved and factors such as extent of private/public care provision. However, the broad aggregation of service types used here should allow transferability to other developed health care systems. Calculation of the eFI once yearly and yearly aggregation of service use and costs may mask transitions to higher levels of frailty and associated increases in service use within the calendar year. However, this pragmatic approach has been used in preference to a mid-year estimate, as measurement error may be introduced whichever calendar cut-point was chosen, and overall trends in both frailty transitions and associated costs are in line with other literature. Finally, due to the highly skewed service use and cost data, it was not possible to account for within-individual correlation in the analyses and also produce cost predictions in a format that was useful for service planning.

### Implications for practice and further research

Estimation of the required workforce to deliver services used by people living with frailty is central to future planning and should be informed by assessment of demand. We intend to extend use of this data to produce demand-led estimates of workforce requirements in different service configurations [40].

Given the increasing number of people living with frailty, which will be further impacted by the continuing effects of austerity, reduced healthy life expectancy and deepening health inequalities exacerbated by the effects of the Severe acute respiratory syndrome coronavirus 2 (SARS-Cov-2) pandemic [41–43], understanding of service use and targeted commissioning of services is essential. Public health prevention strategies are likely to be more cost-effective compared to health service interventions [44] and need to be used in conjunction with direct care to support the long-term sustainability of healthcare systems [45]. However, although the core principles may be clear, many evidence gaps remain as to effectiveness and cost-effectiveness of interventions to change behaviours and modifiable risk factors to achieve better morbidity and mortality outcomes [38], and this is an area for urgent research.

## Conclusions

Frailty has a large impact on service use and costs for people with frailty across adult life. Better preventive management of risk groups, and earlier intervention and prevention of decline across adult life should modify service use and costs. Predictions of service use and the cost of providing additional services in an ageing population are essential to balance the impact of preventive and proactive responsive services to effectively plan appropriate care and optimise resource use.

## Supplementary Material

aa-23-1562-File002_afae010
