# Supplementary material for: Primary and secondary care service use and costs associated with frailty in an ageing population: longitudinal analysis of an English primary care cohort of adults aged 50 and over, 2006–2017
Source: Age Ageing. 2024 Feb 9;53(2):afae010. doi: 10.1093/ageing/afae010 (PMC10857897; doi:10.1093/ageing/afae010)
Supplement: aa-23-1562-File002_afae010 [file aa-23-1562-file002_afae010.docx]

**Primary and secondary care service use and costs associated with frailty in an ageing population: longitudinal analysis of an English primary care cohort of adults aged 50 and over, 2006–2017**

**Appendices**

**Contents:**

Appendix 1. Total primary care service use per calendar year of the cohort

Appendix 2. Total secondary care service use per calendar year of the cohort

Appendix 3 Proportion of costs of primary, secondary and total care by frailty category over the study period

Appendix 4. Predicted adjusted mean costs by frailty category (£)

**Appendix 1. Total primary care service use per calendar year of the cohort**

|  | **Population (n)^1^** | **Type of primary care service use** | | | | |
| --- | --- | --- | --- | --- | --- | --- |
| **Year** |  | **Face-to-face appointments** | **Home visits** | **Telephone triage** | **e-consultations** | **Number of individual prescriptions for medicines** |
| **2006** | 1,104,135 | 7,370,637 | 384,918 | 587,971 | 55 | 28,891,568 |
| **2007** | 1,137,583 | 7,745,118 | 396,894 | 674,454 | 470 | 31,139,738 |
| **2008** | 1,168,955 | 8,147,716 | 401,526 | 800,575 | 763 | 33,695,233 |
| **2009** | 1,198,789 | 8,829,294 | 399,355 | 892,740 | 960 | 36,128,665 |
| **2010** | 1,231,606 | 9,026,016 | 392,703 | 944,429 | 1,360 | 38,433,223 |
| **2011** | 1,266,747 | 9,301,347 | 371,122 | 1,022,779 | 1,667 | 40,600,594 |
| **2012** | 1,302,815 | 9,488,829 | 351,795 | 1,139,451 | 2,701 | 42,665,590 |
| **2013** | 1,345,927 | 9,392,535 | 330,637 | 1,329,383 | 3,749 | 45,067,424 |
| **2014** | 1,382,020 | 9,342,733 | 330,592 | 1,549,521 | 5,261 | 47,226,025 |
| **2015** | 1,425,275 | 9,310,318 | 333,296 | 1,700,580 | 5,728 | 49,432,669 |
| **2016** | 1,461,387 | 9,462,405 | 331,005 | 1,844,310 | 10,882 | 50,854,402 |
| **2017** | 1,489,495 | 9,587,055 | 319,661 | 1,967,474 | 18,493 | 52,339,081 |

^1^ Population as of 1^st^ January

**Appendix 2. Total secondary care service use per calendar year of the cohort**

|  | **Population (n) ^1^** | **Type of secondary care service use** | | | | | | |
| --- | --- | --- | --- | --- | --- | --- | --- | --- |
| **Year** |  | **Outpatient appointments** | **Emergency department attendances** | **Total hospital admissions** | **Elective admissions** | **Unplanned admissions** | **Total days of hospital stay** | **Critical care**  **admissions** |
| **2006** | 1,104,135 | 1,808,701 | NO DATA | 429,727 | 276,275 | 144,611 | 1.966,447 | NO DATA |
| **2007** | 1,137,583 | 1,935,668 | 151,906 | 460,831 | 304,478 | 148,319 | 1,926,362 | NO DATA |
| **2008** | 1,168,955 | 2,179,469 | 234,548 | 498,460 | 335,510 | 158,554 | 1,985,763 | 2,854 |
| **2009** | 1,198,789 | 2,453,640 | 277,158 | 527,911 | 350,164 | 168,974 | 2,042,322 | 4,710 |
| **2010** | 1,231,606 | 2,628,881 | 311,610 | 557,455 | 371,954 | 176,631 | 2,004,159 | 6,139 |
| **2011** | 1,266,747 | 2,752,260 | 335,213 | 576,709 | 387,984 | 180,114 | 1,974,950 | 7,049 |
| **2012** | 1,302,815 | 2,942,468 | 364,643 | 606,217 | 405,736 | 191,759 | 2,080,816 | 7,565 |
| **2013** | 1,345,927 | 3,205,647 | 383,236 | 630,058 | 421,101 | 200,073 | 2,127,694 | 8,216 |
| **2014** | 1,382,020 | 3,541,389 | 407,779 | 674,531 | 453,749 | 211,920 | 2,177,885 | 8,626 |
| **2015** | 1,425,275 | 3,848,590 | 426,926 | 697,853 | 472,240 | 217,116 | 2,183,777 | 8,924 |
| **2016** | 1,461,387 | 4,091,534 | 470,057 | 726,173 | 488,833 | 229,530 | 2,251,645 | 9,147 |
| **2017** | 1,489,495 | 4,218,322 | 479,498 | 740,674 | 497,626 | 235,861 | 2,150,553 | 9,412 |

^1^ Population as of 1^st^ January

**Appendix 3 Proportion of costs of primary, secondary and total care by frailty category over the study period**

**Appendix 4. Predicted adjusted mean costs by frailty category (£)**

|  | **Primary care**  **Mean cost (£)**  **[95% confidence interval]** | **Secondary care**  **Mean cost (£)**  **[95% confidence interval]** | **Total care**  **Mean cost (£)**  **[95% confidence interval]** |
| --- | --- | --- | --- |
| **Frailty category** |  |  |  |
| Fit | 346.51 [346.25 – 346.76] | 612.46 [610.12 – 614.80] | 957.27 [955.16 – 959.39] |
| Mild | 810.94 [810.05 – 811.83] | 1,354.79 [1347.67 – 1,361.92] | 2,158.95 [2,152.16 – 2,165.73] |
| Moderate | 1,136.34 [1,134.14 – 1,138.53] | 2,092.67 [2,073.69 – 2,111.64] | 3,219.83 [3,202.19 – 3,237.47] |
| Severe | 1,517.69 [1,512.56 – 1,522.82] | 2,943.90 [2,897.01 – 2,990.79] | 4,464.23 [4,421.32 – 4,507.15] |
